# Lessons in aging from Myc knockout mouse models

**DOI:** 10.3389/fcell.2023.1244321

**Published:** 2023-08-09

**Authors:** Edward V. Prochownik, Huabo Wang

**Affiliations:** ^1^ Division of Hematology/Oncology, UPMC Children’s Hospital of Pittsburgh, Pittsburgh, PA, United States; ^2^ The Department of Microbiology and Molecular Genetics, UPMC, Pittsburgh, PA, United States; ^3^ The Hillman Cancer Center of UPMC, Pittsburgh, PA, United States; ^4^ The Pittsburgh Liver Research Center, UPMC, Pittsburgh, PA, United States

**Keywords:** cancer, glycolysis, MLX, mitochondria, progeria, ribosomes, ROS, senescence

## Abstract

Despite *MYC* being among the most intensively studied oncogenes, its role in normal development has not been determined as *Myc−/−* mice do not survival beyond mid-gestation. *Myc* ± mice live longer than their wild-type counterparts and are slower to accumulate many age-related phenotypes. However, *Myc* haplo-insufficiency likely conceals other important phenotypes as many high-affinity Myc targets genes continue to be regulated normally. By delaying *Myc* inactivation until after birth it has recently been possible to study the consequences of its near-complete total body loss and thus to infer its normal function. Against expectation, these *“Myc*KO” mice lived significantly longer than control wild-type mice but manifested a marked premature aging phenotype. This seemingly paradoxical behavior was potentially explained by a >3-fold lower lifetime incidence of cancer, normally the most common cause of death in mice and often Myc-driven. *Myc* loss accelerated the accumulation of numerous “Aging Hallmarks”, including the loss of mitochondrial and ribosomal structural and functional integrity, the generation of reactive oxygen species, the acquisition of genotoxic damage, the detrimental rewiring of metabolism and the onset of senescence. In both mice and humans, normal aging in many tissues was accompaniued by the downregulation of Myc and the loss of Myc target gene regulation. Unlike most mouse models of premature aging, which are based on monogenic disorders of DNA damage recognition and repair, the *Myc*KO mouse model directly impacts most Aging Hallmarks and may therefore more faithfully replicate the normal aging process of both mice and humans. It further establishes that the strong association between aging and cancer can be genetically separated and is maintained by a single gene.

## 1 Introduction

### 1.1 The *MYC* oncogene and its role as a transcription factor in cancer

*MYC* bears the distinction of being among the first transforming retroviral oncogenes (v-myc) that was discovered before it cellular counterpart (c-Myc) (Duesberg and Vogt, 1979; Roussel et al., 1979; Sheiness et al., 1980; Hayward et al., 1981; Ramsay et al., 1990). Its long and storied history, combined with its well-documented involvement in many human cancers, provides ample reason as to why it persists after nearly 50 years as being among the most intensely studied of all mammalian oncogenes (Meyer and Penn, 2008). Myc’s widespread role in human cancer pathogenesis also explains why efforts to identify and develop effective inhibitors remain a major priority despite the frustratingly difficult nature of this task (Weber and Hartl, 2023).

**FIGURE 1:**
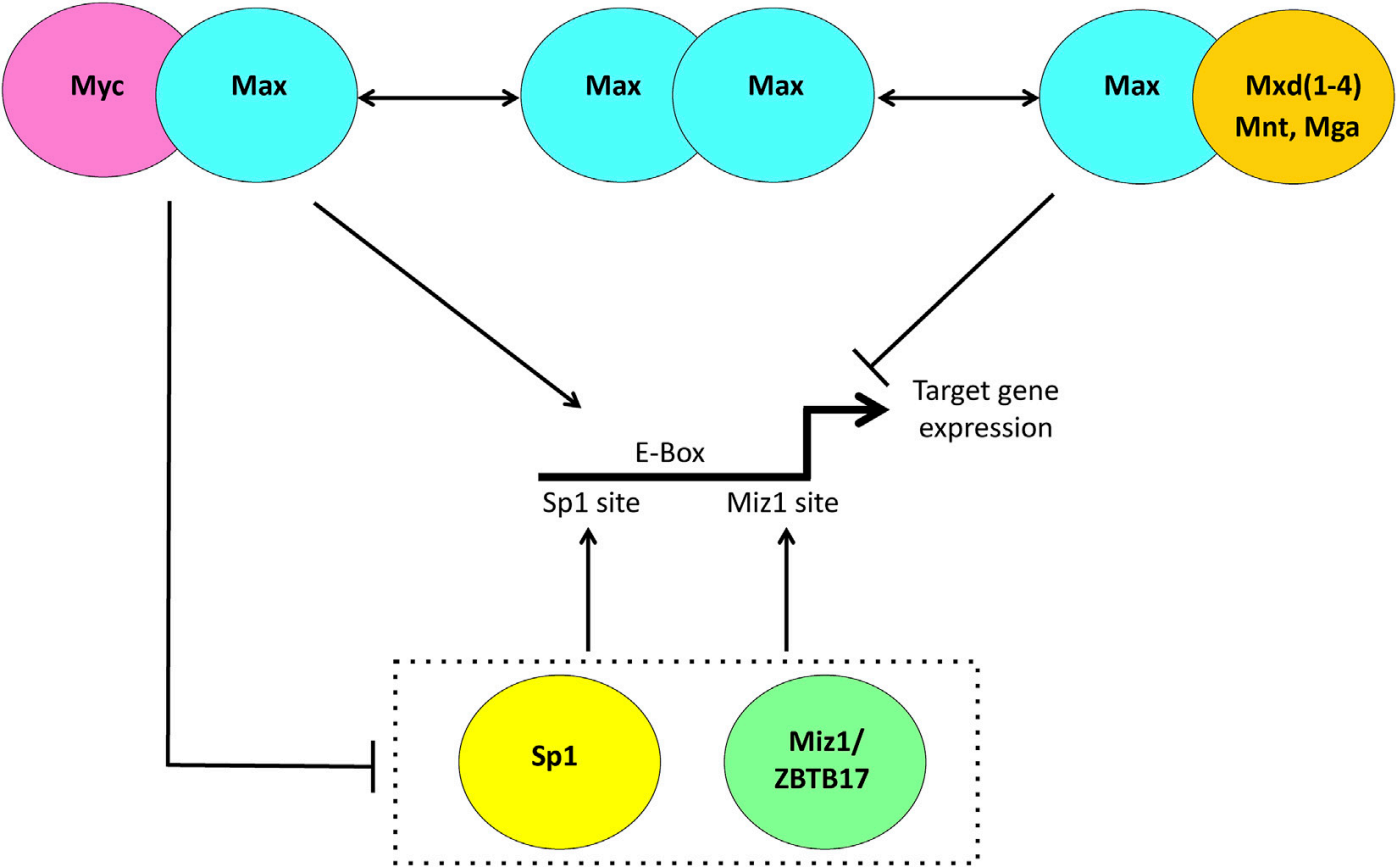
Transcriptional regulation via the Myc Network. When Myc is abundant, such as during proliferation, it heterodimerizes with the bHLH-ZIP protein Max, binds to consensus E box elements, usually located in the proximal promoters of its target genes and facilitates transcription. When Myc levels are low such as in quiescent cells, Max is more likely to heterodimerize with members of the Mxd family, comprised of the related bHLH-ZIP factors Mxd1-4 and the more distantly related Mnt and Mga. These can compete with and displace Myc-Max heterodimers from E boxes and silence gene expression. Negative regulation of Myc target genes is achieved indirectly as a result Myc-Max heterodimers binding to and suppressing the positively-acting transcription factors Sp1/3 and Miz1/ZBTB17.

The Myc protein is a bHLH-ZIP transcription factor that regulates thousands of downstream target genes or perhaps even the entirety of the genome by serving as a more general transcriptional amplifier of gene expression (Eilers and Eisenman, 2008; Nie et al., 2012; Carroll et al., 2018; Patange et al., 2022). Positive regulation is achieved upon Myc’s association with its bHLH-ZIP partner protein, Max, and binding of the heterodimer to consensus “E box” elements that are typically located in the proximal promoters of its direct target genes (Figure 1) (Eilers and Eisenman, 2008; Carroll et al., 2018; Prochownik, 2022). The general consensus is that the extent to which a gene is upregulated by Myc is determined largely, although not exclusively, by several independent and non-mutually exclusive factors. These include the intrinsic affinity of the Myc-Max heterodimer for the target gene’s associated E box (es); the E box’s epigenetic modification; the degree to which neighboring chromatin is itself epigenetically altered and relaxed to allow access of Myc-Max to the E box; the presence of other unrelated factors that may bind nearby and hinder or promote Myc-Max binding and the extent to which Myc-Max must compete with other E box-binding transcription factors including those between Max and members of the Mxd family, which actively oppose Myc by transcriptionally suppressing its target genes (Figure 1) (Prendergast and Ziff, 1991; Conacci-Sorrell et al., 2014; Dolezal et al., 2017; Carroll et al., 2018; Prochownik, 2022; Prochownik and Wang, 2022). Collectively the integrated interplay and cooperation among these various factors serve to define the “functional affinity” of a binding site. This operative definition allows for changes in these affinities in ways that reflect different cell types, states of proliferation or differentiation and the dynamic nature of intracellular conditions and cues.

Among the most critical determinants of whether and to what extent Myc will upregulate a target gene is the absolute level of Myc protein itself (Dolezal et al., 2017; Wang et al., 2022b). This led to the concept of “physiologic” and “pathologic” targets (Fernandez et al., 2003; Soucek and Evan, 2010; Dolezal et al., 2017; Prochownik, 2022; Prochownik and Wang, 2022) with the former being defined as genes that respond to levels of Myc that can be achieved in normal cells during, for example, periods of log-phase growth. The binding sites in such targets might therefore be considered as being of moderate-high affinity based on the above definition. In contrast, pathologic targets bind and/or respond to the high Myc levels that are only observed in tumors or in untransformed cells with experimentally enforced Myc over-expression (Coller et al., 2000; Nesbit et al., 2000; Zeller et al., 2006; Sabo et al., 2014). These may include previous physiologic targets that are now induced to even higher levels or targets that bind Myc-Max and respond to it only when it is over-expressed. The binding sites in these targets might therefore be considered as being low-affinity. The relevance of pathologic targets is dramatically underscored *in vivo* where high-level conditional Myc induction can rapidly induce aggressive tumors and the expression of unique transcriptomes whereas subsequent Myc inactivation causes complete tumor regression and transcriptomic normalization even before the tumor itself shows any objective gross or histologic response (Karlsson et al., 2003; Shachaf et al., 2004; Wu et al., 2007; Dolezal et al., 2017). Despite these distinctions, it is likely that both physiologic and pathologic targets contribute to transformation (Soucek and Evan, 2010; Prochownik, 2022; Prochownik and Wang, 2022).

In contrast to Myc’s positive transcriptional regulation, an equal or somewhat smaller fraction of its target gene repertoire is negatively regulated via more indirect mechanisms. This is accomplished by interactions between Myc-Max heterodimers and the transcription factors Miz1, Sp1 and Sp3, thereby preventing the upregulation of target genes bearing Miz1 and/or Sp1 sites in their promoters (Figure 1) (Gartel et al., 2001; Gartel and Shchors, 2003; Eilers and Eisenman, 2008; Herkert and Eilers, 2010). While there is less direct evidence that Myc’s negative targets are subject to the same types of physiologic and pathologic regulation as positive targets, this does appear to be the case as evidenced by data showing much larger numbers of Miz1 and Sp1 binding sites being co-occupied by Myc and an expansion of negative target gene responses during peaks of high physiologic or pathologic Myc expression (Encode Project Consortium, 2012; Diehl and Boyle, 2016; Wang et al., 2018; Wang et al., 2022a).

## 2 Myc target genes and the consequences of Myc inhibition *in vitro* and *in vivo*

The proteins encoded by Myc target genes can be broadly classified into several general functional categories (Zeller et al., 2006; Kim et al., 2008; Anczukow and Krainer, 2015; Wang et al., 2022a; Wang et al., 2022b; Prochownik, 2022). Their duties include promoting the cell cycle; overseeing the structure and function of mitochondria; regulating translation, notably, the synthesis of ribosomal subunits, tRNA, rRNAs and translation/initiation factors; coordinating non-mitochondrial metabolic pathways, particularly glycolysis, glutaminolysis, and lipid and nucleotide biosynthesis; the control of mRNA splicing and the recognition and repair of various types of DNA damage (Grandori et al., 2000; Felton-Edkins et al., 2003; Grandori et al., 2005; Li et al., 2005; Gomez-Roman et al., 2006; Mannava et al., 2008; Dang, 2010; van Riggelen et al., 2010; Dang, 2011; Carroll et al., 2018; Singh et al., 2019; Singh et al., 2021; Wang et al., 2022a; Wang et al., 2022b; Prochownik, 2022). In primary murine embryonic fibroblasts (MEFs), which undergo immediate cell cycle arrest in response to *Myc* inactivation, 2 additional sets of genes related to aging and senescence have also been recently identified (Wang et al., 2022b).

Regardless of whether *MYC* is silenced genetically or pharmacologically, its inhibition *in vitro* is almost always associated with an immediate cessation of proliferation that usually coincides with G_0_/G_1_ arrest, although in cancer cells this may occur in other stages of the cell cycle or even in all stages simultaneously (Trumpp et al., 2001; Huang et al., 2006; Wang et al., 2008; von Bueren et al., 2009; Wang et al., 2015; Scognamiglio et al., 2016; Wang et al., 2022b). *In vivo,* total deletion of *Myc* in the embryo is uniformly lethal at ∼e10.5 (Davis et al., 1993). Embryos of *Myc* hypomorphs engineered to express progressively lower Myc levels show dose-related reductions in body size as Myc levels decline and MEFs derived from these mice also show a gradual, and eventual total loss of proliferative capacity (Trumpp et al., 2001). Examination of individual cell populations from these mice showed their overall smaller organ size to be due to reductions in the total cellular content rather than decreases in cell size (Trumpp et al., 2001). Similar defects have been demonstrated in isolated Myc-depleted T lymphocytes whose activation did not affect cell growth but did severely impair their ability to proliferate (Wang et al., 2011).

In contrast, and for unknown reasons, transient inhibition of Myc *in vivo* in older mice rarely has such dramatic effects. For example, body-wide induction in adult mice of the dominant-negative Myc inhibitor known as OmoMyc was entirely compatible with survival but did cause mild and transient bone marrow hypoplasia and flattening of the intestinal mucosa, both of which were reversible despite continued Myc suppression (Soucek et al., 2008). Neither the degree to which OmoMyc inhibited the function of endogenous Myc nor any long-term follow up of these mice was reported. On the other hand, Myc inhibition was sufficient enough to promote the regression of pre-existing Ras-driven lung tumors indicating that certain neoplasms and their responsible oncogenes can display a high Myc-dependency both *in vivo* and *in vitro* (Sklar et al., 1991; Karlsson et al., 2003; Shachaf et al., 2004; Wu et al., 2007; Soucek et al., 2008; Dolezal et al., 2017). In contrast to these findings, the hepatocyte-specific elimination of Myc did not alter the time needed for mice to regenerate a normal liver mass following 2/3rd partial hepatectomy (Baena et al., 2005; Li et al., 2006; Sanders et al., 2012). Concerned that this procedure did not provide a sufficiently strong or lengthy proliferative demand, Edmunds et al. employed the “FAH” mouse model of hereditary tyrosinemia to show that the long-term ability of transplanted wild type and *Myc−/−* donor hepatocytes to repopulate the liver, replace the diseased hepatocyte population and cure the recipient mice were in all cases equivalent (Overturf et al., 1996; Edmunds et al., 2016). Indeed, not even subtle differences were observed in the long-term repopulation by the 2 donor hepatocyte populations when they were allowed to compete in the same recipient.

The above findings raised questions as to Myc’s role in initiating and/or supporting neoplastic hepatocytes proliferation mediated by other oncogenes. This was examined in a mouse model of hepatoblastoma (HB) in which tumors could be rapidly and efficiently induced via the hydrodynamic tail vein-mediated delivery of Sleeping Beauty plasmids encoding mutant forms of β-catenin and the Hippo pathway effector YAP (Tao et al., 2014; Bell et al., 2017; Zhang et al., 2019). When these vectors were delivered to the previously mentioned mice lacking *Myc* in their hepatocytes, tumor initiation remained at 100% although the ensuing growth was markedly slowed and survival was prolonged (Wang et al., 2016). It was concluded that, at least within this model neoplastic framework, endogenous Myc was not necessary to initiate tumorigenesis but was necessary to maintain maximal tumor growth rates. This was supported by the finding that, relative to *Myc+/+* HBs, *Myc−/−* HBs expressed lower levels of transcripts encoding proteins involved in the structure and function of mitochondria and the translational machinery, including most ribosomal proteins and many translation factors. Consistent with a less pronounced Warburg effect, *Myc−/−* tumors also showed an attenuated induction of transcripts encoding glycolytic enzymes and lower levels of fatty acid β-oxidation (FAO). Perhaps as testimony to a less pronounced upregulation of Myc target genes, global histone H3K9 acetylation was reduced in tumors from *Myc−/−* mice (Martinato et al., 2008; Wang et al., 2016). Together with the previously mentioned findings, these studies provided a more nuanced role for Myc in both normal and neoplastic development and suggested that Myc’s role in the generation of these tumors was not to participate in tumor initiation (or at least in its most critical aspects) but rather to provide the necessary translational and metabolic support needed to achieve maximal rates of tumor growth.

## 3 Embryonal *Myc* heterozygosity extends lifespan and improves healthspan

The embryonic lethality of *Myc−/−* mice (Davis et al., 1993; Trumpp et al., 2001; Dubois et al., 2008) has until recently precluded an evaluation of Myc’s role in growth and development beyond mid-gestation. Attempting to make the best of a bad situation, Hofmann et al. studied the life-long consequences of mice that had been rendered heterozygous for *Myc* (*Myc* ± mice) at the time of conception (Hofmann et al., 2015). These mice displayed several unanticipated phenotypes. First, and perhaps most importantly, they lived on average about 15% longer than their *Myc+/+* (wild-type) counterparts with significant differences being noted between the sexes (20.9% longer for females and 10.7% longer for males). Like previously described *Myc* hypomorphs, *Myc* ± mice were also smaller at the time of birth and remained so, with the proportional mass of their individual organs being reduced accordingly so as to maintain the same relationship to total body mass as seen in wild-type mice. Both groups of mice showed a similar incidence of cancer during their lifetimes, the vast majority of which were lymphomas as has been reported in most strains of mice (Ward, 2006; Snyder et al., 2016). Thus, the increased longevity of *Myc* ± mice did not appear to be related to a lower cancer incidence, although they were reported to have smaller tumors with less extensive spread, thus perhaps reflecting the experience of Wang et al. in generating HBs in *Myc−/−* hepatocytes.

Relatively few gene expression changes were noted in the 3 tissues that were examined in both young and old *Myc* ± mice by microarray analysis, namely, liver, skeletal muscle and white adipose tissue (Hofmann et al., 2015). Indeed, the gene expression differences that were attributable to the 50% loss of Myc expression were ∼10-fold fewer in number than those associated with aging. In retrospect this is perhaps not surprising given that, as discussed above, the most functionally important Myc target genes in non-neoplastic tissues might be expected to be those with the highest affinity E boxes and thus unlikely to be particularly impacted by a 2-fold loss in Myc expression. However, among the most significant and important functional classes of genes to be downregulated in *Myc* ± tissues based on gene set enrichment were those related to ribosome biogenesis and rRNAs (Hofmann et al., 2015).

Despite the relatively modest impact on Myc target gene expression, Hoffmann et al. identified a number of phenotypic changes in *Myc* ± mice that, in addition to the lifespan extension, were consistent with delayed aging and an extended health span. These included lower levels of age-related cardiac fibrosis, osteoporosis, hepatic lipid accumulation, serum cholesterol and loss of motor coordination as measured by rotarod testing. The age-related exhaustion of long-term hematopoietic stem cells was also delayed in *Myc* ± mice (Hofmann et al., 2015). Importantly, neither overall body adiposity not the proportion of senescent cells were impacted in *Myc* ± animals. Double-stranded DNA breaks (DSBs), as measured by the accumulation of 53BP1 foci in livers also increased equally with aging in the livers of both wild-type and *Myc* ± mice. This suggested that the reduced rate of aging of *Myc* ± mice could not be attributed to a lower rate of DNA damage (or at least of DSBs).

Metabolic cage studies pointed to *Myc* ± mice as having significantly higher metabolic rates, as measured by total oxygen consumption (Nie et al., 2015). Although CO_2_ production rates were not reported, the implication of these studies was that the respiratory exchange ratio (RER), as determined by the VCO_2_/VO_2_ ratio, was lower and that *Myc* ± mice were more reliant on fatty acid oxidation as an energy source. Finally, consistent with the previously mentioned reduction in transcripts related to ribosomes and rRNAs, Hoffman et al. found evidence for a decreased rate of total liver protein synthesis as measured by the *in vivo* incorporation of radio-labeled phenylalanine (Hofmann et al., 2015).

## 4 Post-natal deletion of *Myc* is associated with premature aging, increased lifespan and a lower cancer incidence

Not all findings in *Myc* ± mice pointed to a slowing of the aging process and an overall healthier lifespan (Hofmann et al., 2015). For example, the reduced rates of ribosomal biogenesis, rRNA production and translation described by Hofmann et al. are actually common properties of aging whereas the presumptive increased reliance on FAO as an energy source might be indicative of another age-related phenomenon, namely glucose intolerance and the switch to fatty acids as an alternate energy source (Chang and Halter, 2003; D'Aquila et al., 2017; Gonskikh and Polacek, 2017; Woodward and Shirokikh, 2021). Although FAO normally declines with age (Toth and Tchernof, 2000), this might not be the case with *Myc* ± mice whose defects in glycolysis and mitochondrial structure and function might have forced an unnatural over-reliance on FAO, which is a more efficient means of energy extraction (Li et al., 2005; Prochownik, 2022).

As mentioned above, the most critical Myc target genes may well be those with the highest affinity binding sites that would be impacted minimally, if at all, by a 50% decline in Myc levels. Thus the phenotypes described by Hofmann et al. (Hofmann et al., 2015) may well represent only the “tip of the iceberg” and may even be quite different from those associated with a more thorough loss of Myc expression. Therefore, in response to these considerations, Wang et al. performed 2 studies in parallel, with each informing the other, that sought to characterize the long-term consequences of a more extensive and potentially more consequential body-wide knockout of Myc (Wang et al., 2022b; Wang et al., 2023). The second study in particular asked whether it was possible to achieve a more extensive loss of *Myc in vivo* while avoiding the lethality associated with *Myc−/−* embryos (Davis et al., 1993; Trumpp et al., 2001). The question was partly motivated by the findings that OmoMyc induction in adult mice had been previously found to be compatible with at least short-term survival as well as by the observation that the viability of *Myc−/−* embryos could be extended by 2 days if Myc expression was preserved in the placenta (Dubois et al., 2008; Soucek et al., 2008). Concerns as to whether high-levels of *Myc* knockout could be achieved without compromising viability remained however given that both OmoMyc-expressing mice and *Myc−/−* embryos showed marked hematopoietic compromise (Trumpp et al., 2001; Dubois et al., 2008; Soucek et al., 2008).

In both studies undertaken by Wang et al., mice bearing 2 “floxed” *Myc* alleles were crossed with a strain bearing a CreER transgene under the control of the ubiquitously expressed Rosa26 promoter (Wang et al., 2022b; Wang et al., 2023). In the first study, e14-16 MEFs were isolated from these mice, expanded for 2-3 passages and then exposed to 4-hydroxytamoxifenn (4OHT) for 7–10 days. This resulted in a >95% excision of the *Myc* gene, a comparable loss of Myc protein expression and proliferative arrest in G_0_/G_1_, which, over the ensuing week became permanently consolidated in G_2_/M. These “*Myc*KO” cells appeared to be larger, flatter and less spindle-shaped, all of which mimicked the previously noted characteristics of senescent primary fibroblasts and of a unique immortalized *Myc−/−* rat fibroblast line that grows at ∼20% the rate of its *Myc* ± counterparts (Mateyak et al., 1997; Zhao and Darzynkiewicz, 2013; Kumari and Jat, 2021).

Growth-arrested primary *Myc*KO MEFs shared additional features with *Myc−/−* rat fibroblasts and cancer cell lines in which Myc function was inhibited genetically or by structurally diverse small molecule Myc inhibitors (Graves et al., 2012). These included increases in mitochondrial-derived reactive oxygen species (ROS) and neutral lipid content suggesting that *Myc*KO MEFs contained functionally defective mitochondria. However, unlike the above cancer cell lines and *Myc−/−* rat fibroblasts, which cannot sustain adequate ATP levels, an ∼2-fold increase in mitochondrial mass observed in *Myc*KO MEFs was postulated to represent a means of compensating for energy generating defects as often occurs in association with aging, senescence and various mitochondrial stresses and diseases (Trifunovic et al., 2004; Barrientos, 2012; Miwa et al., 2022). Thus, rather than completely mimicking the properties of immortalized *Myc−/−* fibroblasts, *Myc*KO MEFs more closely recapitulated the behaviors of aging and/or senescence primary fibroblasts, whose proliferation declines and eventually ceases with continued *in vitro* passage and whose mitochondrial content increases in parallel (Hayflick, 1974; Korolchuk et al., 2017; Popay et al., 2021; Martini and Passos, 2023). Like senescent cells, growth-arrested primary *Myc*KO MEFs also showed increased lysosomal content and glucose uptake, higher levels of senescence-associated β-galactosidase and decreased translation as measured by puromycin incorporation into elongating polypeptide chains (Bittles and Harper, 1984; Sharpless and Sherr, 2015; Payea et al., 2021; Popay et al., 2021; Wang et al., 2022b).

RNAseq performed on WT primary and *Myc*KO MEFs within 10 days of *Myc* excision revealed >4300 gene expression differences, about equally divided between up- and downregulated transcripts and with nearly 2/3^rds^ of them being encoded by previously identified direct Myc target genes (Wang et al., 2022b). Gene set enrichment analysis (GSEA) categorized these into a small number of functionally-related categories that were consistent with some but not all previously described Myc target gene classifications. These functions included those dedicated to mitochondrial and ribosomal structure and function, cell cycle regulation, aging, senescence and the recognition and repair of multiple types of DNA damage. In follow-up to this latter observation, immuno-staining showed that *Myc*KO primary MEFs expressed higher levels of the DNA damage recognition and response proteins p53, 53BP1, γ-H2AX, RAD51 and Ku80 while also showing increased staining in a TUNEL assay, which, like γ-H2AX staining, identifies DSBs. Furthermore, whereas treatment of WT MEFs with the DSB-inducing chemotherapeutic drug etoposide elicited a coordinated response of the above factors, the response in *Myc*KO MEFs was suppressed and dysregulated. These findings suggested that *Myc*KO MEFs displayed more evidence of baseline genotoxic stress, not only as a result of their increased ROS production but also due to their inability to properly marshal and sustain a well-regulated DNA damage response.

While aging and senescence are thought to be at least partially driven by the accumulation of DNA damage, it is also true that old and/or senescent cells are less capable of DNA damage repair and therefore generate genotoxic lesions at faster rates than younger cells and maintain them longer (Chen et al., 2007; Collin et al., 2018; Schumacher et al., 2021; Yousefzadeh et al., 2021). To test the idea that *Myc*KO cells might be more prone to DNA damage, Wang et al. took advantage of the unexpected finding that SV40 T antigen-immortalized *Myc*KO MEFs could escape proliferative arrest in response to *Myc* inactivation and continue to replicate at about half the normal rate (Wang et al., 2022b). The RNAseq profiles of these cells also showed that they retained evidence of numerous DNA damage recognition and repair pathway defects. As a result, these cells were significantly more resistant than wild-type immortalized MEFs to genotoxic insults that, in addition to DSBs, included single-stranded breaks, oxidative base lesions and both inter- and intra-strand cross-links. Wang et al. contrasted this seemingly paradoxical behavior to that associated with monogenic disorders of DNA repair such as Fanconi’s anemia and xeroderma pigmentosum, which are exquisitely sensitive to DNA damage (Black, 2016; Taylor et al., 2019). They suggested that the non-repairable lesions associated with these inherited conditions initiate a robust apoptotic response since the pathways that mediate this remain intact. In contrast, the multiple defects in *Myc*KO MEFs are so extensive that any new DNA damage is neither recognized, repaired nor able to elicit a coordinated apoptotic response. A similar loss of sensitivity to cis-platinum and etoposide has been observed in medulloblastoma cell lines following the siRNA-mediated knockdown of Myc (von Bueren et al., 2009).

The second study reported by Wang et al. utilized the above-described *Myc* ^*loxP/loxP*^ x Rosa26-CreER mouse strain in which individuals of both sexes were treated with 5 daily injections of tamoxifen beginning on the day of weaning (Wang et al., 2023). The timing of these injections was critical as preliminary studies had shown that treating younger mice or those weighing <15–16 grams was associated with a high incidence of fatal aplastic anemia. qPCR and qRT-PCR analysis performed with over a dozen tissues from treated mice showed that *Myc* gene excision frequencies exceeded 75%–95% in nearly all tissues with a notable exception being brain where it was 40%–60%. The observation that these “*Myc*KO” mice not only survived the major cause of embryonic mortality (Davis et al., 1993; Trumpp et al., 2001; Dubois et al., 2008) but also remained seemingly healthy allowed for other previous findings to be confirmed and further extended. For example, while >90% of mice survived and eventually normalized their peripheral counts, bone marrows remained hypoplastic and, by 4–5 months of age, resembled those of middle-aged mice (∼50% cellularity). Like the OmoMyc-treated mice described by Soucek et al. *Myc*KO mice also displayed transient intestinal epithelial flattening and loss of crypt structure (Soucek et al., 2008; Wang et al., 2023). Yet, despite these obvious morphological changes, the mice showed no evidence of steatorrhea or failure to thrive indicating that the changes had minimal physiologic impact. These findings, as well as well as additional ones described below, are summarized in Table 1 where they are compared and contrasted with those from *Myc−/−* embryos and *Myc* ± mice (Davis et al., 1993; Trumpp et al., 2001; Dubois et al., 2008; Hofmann et al., 2015).

**TABLE 1:** Notable phenotypic differences among mice with varying degrees of myc inactivation vs. WT controls.

	Genotype (references)		
	*Myc*−/− Davis et al. (1993); Trumpp et al. (2001); Dubois et al. (2008)	*Myc* ± Hofmann et al. (2015)	*Myc*KO Wang et al. (2023)
Timing of knockout	Embryonal	Embryonal	Post-natal
Extent of knockout	100%	100%	Variable (∼70%–100%)
Lifespan	embryonal lethal	Extended	Extended
Lifetime cancer incidence	ND^a^	Normal	3.4-fold lower
Size of mice	Reduced	Reduced	Normal
Major organ structural defects	placenta, BM^b^, vasculature	None	BM, intestine Fat:lean mass ratio
Fat:lean mass ratio	ND	ND	Prematurely Increased
Hepatic steatosis	ND	No	Yes
Alopecia	ND	No	Yes
Achromotricia	ND	No	Yes
Hyperkeratinization	ND	ND	Yes
Overall strength, endurance, balance	ND	Better	Worse
Glucose tolerance	ND	ND	T2D-like GTT
Serum cholesterol	ND	Reduced	ND
Cardiac fibrosis	ND	Less severe	ND
Osteoporosis	ND	Less severe	ND
CD4:CD8 T cell ratio	ND	Less pronounced decline	ND
Increased genotoxic stress	ND	No	Yes
Oxygen consumption	ND	Increased: day time + night time	Increased: night time only
Rate of protein translation	ND	Decreased	Decreased
Energy deficit	ND	Yes	ND

^a^ ND, not determined.

^b^ BM, bone marrow.

Although the body weights of *Myc*KO and wild-type mice were initially indistinguishable, the former began to acquire significantly higher fat:lean mass ratios after 5–6 months such that by 10 months of age these ratios resembled those of ∼20 month old wild-type mice (Pappas and Nagy, 2019). Both groups then began to lose weight and to reduce their fat:lean mass ratios at similar rates, although both parameters remained high in *Myc*KO mice for the remainder of their lives. *Myc*KO mice also developed premature graying and loss of fur beginning at 3–5 months of age that resembled, albeit to a lesser degree, the phenotype of old mice or those with melanocyte-specific embryonal excision of *Myc* (Pshenichnaya et al., 2012). Skin samples from the alopecic regions showed epidermal thickening, hyperkeratinization and focal peri-follicular staining for senescence-associated β-galactosidase.

Testing of *Myc*KO mice for strength, fitness and coordination showed them to be generally inferior to age-matched wild-type mice, although these differences became noticeable at different times throughout life. In older *Myc*KO females (∼20 months), even normal, diurnal ambulatory activity was reduced.

A number of metabolic abnormalities consistent with premature aging also distinguished wild-type and *Myc*KO mice. Nonalcoholic fatty liver disease (NAFLD or hepatic steatosis) commonly accompanies aging, particularly in the face of co-existing conditions such as dyslipidemia, obesity and insulin resistance (Honma et al., 2011; Bertolotti et al., 2014). Although a NAFLD-like picture was previously identified in mice with *Myc−/−* hepatocytes, neither its maximal severity nor its life-long consequences were evaluated (Edmunds et al., 2016). Wang et al. confirmed that the degree of NAFLD in 5 month old *Myc*KO mice, as measured by neutral lipid and triglyceride content, was 3-4-fold higher than that of comparably aged wild-type mice and matched that of 22 month old individuals from the latter cohort (Wang et al., 2023). Thus, although the maximal levels of lipid that were accumulated by *Myc*KO mice never exceeded the highest levels attained in the oldest wild-type mice, the rate of accumulation was more rapid.

Metabolic cage studies were also performed during the lifetimes of wild-type and *Myc*KO mice while being maintained on normal diets, during fasting and after re-feeding with either normal or high-fat diets. Young wild-type mice tended to possess very high nocturnal respiratory exchange ratios (RERs) indicating that, during active feeding, they were almost totally reliant on glucose as an energy source. RERs exceeding 1.0 were observed in some cases and indicated that these mice were engaged in high levels of fatty acid synthesis, which is commonly observed in juvenile animals undergoing rapid growth (Bruss et al., 2010; Houtkooper et al., 2011). In contrast, *Myc*KO mice demonstrated a significantly greater reliance on FAO and their RERs never exceeded 1.0. These findings were interpreted as indicating one or more of at least 3 non-mutually conditions. First, the absence of Myc may have reduced the animals’ ability to metabolize glucose since Myc positively regulates glycolysis (Dang, 2010; Dang, 2011; Stine et al., 2015; Prochownik, 2022). Second, it may also have impaired the efficiency of mitochondria, making them more reliant on FAO to maintain normal energy levels as had been described in *Myc*KO MEFs and other Myc-compromised cells (Wang et al., 2015; Wang et al., 2022a; Wang et al., 2022b). Increased FAO dependency may also explain the neutral lipid accumulation of *Myc*KO mice, MEFs and other cells with compromised Myc function, in which energy-rich fatty acids are taken up in excess of what is needed to maintain energy stores with the difference being stored (Edmunds et al., 2016). Other ways to explain the greater reliance of *Myc*KO cells on FAO include a reduced supply of glycolytically-derived pyruvate for the TCA cycle and/or its diversion into other, non-acetyl coenzyme A-generating pathways (Stine et al., 2015; Prochownik and Wang, 2021). Third, younger *Myc*KO animals may have either already aged beyond the point where increased fatty acid synthesis would have been observed and/or may have compromised Myc-regulated fatty acid synthetic function (Morrish et al., 2010; Singh et al., 2021). Irrespective of cause(s), the RERs of younger *Myc*KO mice tended to more closely mimic those of older wild-type mice with the differences between the 2 groups becoming more erratic and tending to converge as the 2 cohorts aged.

Consistent with their high utilization of fatty acids as an energy source, but also indicating that they may be insulin resistant, *Myc*KO mice were mildly ketotic although fasting glucose and lactate levels were normal. Glucose tolerance testing and the quantification of peripheral insulin levels in *Myc*KO mice showed that they resembled those associated with Type 2 diabetes, with exaggerated hyperglycemia and hyperinsulinemia in response to a glucose challenge. However, these defects became progressively less pronounced with aging, thus indicating that *Myc*KO mice metabolically adapted in a Myc-independent manner while also reflecting their age-dependent tendency toward RER normalization.

Mitochondrial structural and functional compromise as a result of Myc’s loss could explain the above-discussed metabolic abnormalities and would be consistent with the previously documented MEF results (Wang et al., 2022b). The examination of partially purified mitochondria from age-matched wild-type and *Myc*KO livers and adipose tissues showed that, even when pyruvate was non-rate-limiting, the oxygen consumption rates of the latter were blunted, thus strongly suggesting a defective in Complex I function with Complex II responses to succinate being similar in wild-type and *Myc*KO tissues (Wang et al., 2023).

The transport of free fatty acids across the outer mitochondrial membrane requires that they first be converted to fatty acyl-CoAs and then conjugated to carnitine via the rate-limiting enzyme carnitine palmitoyl transferase I (CPTI). They are then transported across the inner mitochondrial membrane, re-transformed via CPTII into a fatty acyl coenzyme A in the matrix and enter the FAO pathway (El-Gharbawy and Vockley, 2018). Complex I defects and the ensuing inefficient oxidation of long chain fatty acids are associated with elevated serum levels of 3-hydroxy-C14-carnitine (C14-OH), which is used as a clinical marker of these disorders (El-Gharbawy and Vockley, 2018). Wang et al. measured the serum levels of 51 acyl carnitines by mass spectrometry (MS) and indeed were able to document significant elevations of C14-OH in 5 month old *Myc*KO mice. As these mice aged, C14-OH levels normalized but were replaced by 12 new changes mostly involving higher levels of longer chain serum acylcarnitines (Wang et al., 2023). This suggested a progressive loss of normal FAO that is observed in aging humans with Type 2 diabetes (Mihalik et al., 2010). It was suggested that the normalization of C14-OH in older *Myc*KO mice cohort resulted from a reduced C14 pool due to the accumulation of the longer chain fatty acyl CoA precursors and their defective oxidation to shorter chain acylcarnitines. Collectively, the findings were consistent with the previous ones indicating that *Myc*KO animals were more insulin-resistant, more dependent on FAO and prematurely developed NAFLD. Interestingly, although the above findings indicated some normalization of the mitochondrial defects that were initially observed in the youngest *Myc*KO mice, older mice from this cohort were noted to have elevated levels of C5 carnitine, which is generally considered as being diagnostic of errors in branched chain amino acid (BCAA) catabolism (Gibson et al., 1994). This suggested that, as *Myc*KO mice aged, their mitochondrial defects worsened and/or broadened so as to increase their utilization of valine, leucine and isoleucine as alternate energy sources. Indeed, a comparison of RNAseq data from the livers of 5 and 20 month old *Myc*KO mice showed enrichment for gene sets involved in FAO at both ages and BCAA catabolism in the older group, thus mirroring the results of serum MS-based measurements and findings from aging humans with Type 2 diabetes (Mihalik et al., 2010).

Perhaps the most unexpected finding from the above studies was that, despite the *Myc*KO mice displaying so many attributes of premature aging, they actually lived significantly longer than the wild-type cohort (median survival 32.7 months vs. 28.3, *p* = 1.2 × 10^−7^) with the longevity difference being particularly notable among females (median survival 33.5 months vs. 27.3 months, *p* = 1.5 × 10^−8^). Indeed, these findings were virtually identical to those of *Myc* ± mice (Hofmann et al., 2015). Careful documentation of the various pathologies observed at the time of death revealed *Myc*KO mice to have a more than 3-fold lower incidence of cancer during their lifetimes but with no change in the tumor spectrum, which was largely comprised of high-grade lymphomas (Ward, 2006; Snyder et al., 2016; Wang et al., 2023). Analysis of lymphomas from 3 *Myc*KO mice showed that Myc re-expression could be detected in at least 2 cases and that the *Myc* gene was intact or even modestly amplified in all 3. These results were interpreted as indicating that the rare tumors arising in *Myc*KO mice likely originated from a small minority population of bone marrow cells that failed to completely excise the *Myc* gene during the initial period of tamoxifen treatment (Wang et al., 2023). It also emphasized that, unlike previously described *Myc*−/− and *Myc* ± mice that were generated at the time of fertilization, all *Myc*KO tissues were almost certainly genetically mosaic, with varying proportions of *Myc+/+, Myc* ± and *Myc−/−* genotypes.

Additional RNAseq studies were performed on liver, skeletal muscle and abdominal white adipose tissues from 5 to 20 month old wild-type and MycKO mice in order to obtain both a broad overview of the genes under Myc’s purview in each of these tissues and an appreciation for how these responded to aging relative to those expressed in wild-type tissues. These tissues were chosen because of previously reported Myc-related changes and because they alter their gene expression profiles during normal aging (Short et al., 2005; Tchkonia et al., 2010; Honma et al., 2011; Hofmann et al., 2015; Uchitomi et al., 2019). The results, which largely agreed with those previously documented in MEFs (Wang et al., 2022b), showed enrichment for at least 7 categories of genes that pointed to their functions being coordinately downregulated and/or compromised in tissues from young *Myc*KO tissues and in aging tissues from both wild-type and *Myc*KO mice.

Transcripts encoding proteins with roles in ribosomal and mitochondrial structure and function tended to be prominently downregulated in younger *Myc*KO tissues and older wild-type tissues although the exact identities of the individual genes and their degree of dysregulation differed in tissue-specific ways (Kim et al., 2008; Edmunds et al., 2016; D'Aquila et al., 2017; Dolezal et al., 2017; Wang et al., 2022b; Wang et al., 2023). Consistent with these findings as well as previous ones pointing to mitochondrial dysfunction in Myc-compromised cells and tissues (Graves et al., 2012; Wang et al., 2016; Wang et al., 2022a; Wang et al., 2022b), a third category of gene sets with roles in the response to oxidative stress was noted to be mostly upregulated in *Myc*KO issues. This was consistent with the previously mentioned increased ROS production resulting from Complex I dysfunction, preference for the use of fatty acids as a source of energy and the increase in mitochondrial mass in at least some tissues (Wang et al., 2022a; Wang et al., 2022b).

Two additional and related gene set categories whose directions of regulation were largely consistent with the premature aging phenotypes of *Myc*KO mice were those specifically associated with aging and senescence. Specifically, members of a 79 member gene set previously shown to be nearly universally dysregulated in response to aging in both mice and humans were largely expressed in opposite directions in wild-type and *Myc*KO mice, with the overall signature pointing to an “older” profile in livers and adipose tissues from the latter group. Several large gene sets previously identified as being enriched in tissues of patients with Type 1 and 2 diabetes, were also dysregulated in all 3 tissues of younger *Myc*KO mice in ways that would have been expected for individuals with these conditions and consistent with the previously documented Type 2 diabetes-like insulin resistance of these animals. In contrast, gene sets found to be enriched in patients with cancer were regulated in opposite ways in young wild-type and *Myc*KO mice with the latter being consistent with the lower life time cancer incidence associated with this group.

The sixth functional category of gene sets that was selectively enriched in young *Myc*KO mouse tissues pertained to DNA damage recognition and its repair, with the directions of dysregulation tending to reflect those seen previously in *Myc*KO MEFs which, as mentioned above, showed much higher levels of ongoing DNA damage despite being highly resistant to a wide variety of genotoxic agents (Wang et al., 2022b). Like *Myc*KO MEF, *Myc*KO livers demonstrated much higher levels of DSBs as documented by immuno-histochemical staining for γ-H2AX (Wang et al., 2023). These studies established that the dysregulation of genes associated with premature aging syndromes due to defective DNA repair pathways were recapitulated in *Myc*KO mice only on a much larger scale.

The final category of gene sets that was significantly enriched between wild-type and *Myc*KO tissues, although only in the liver, pertained to splicing and mRNA processing that includes maturation steps such as intron-exon recognition, lariat formation and excision and exon-exon ligation (Yan et al., 2019). A search for an excess of incorrectly or incompletely spliced transcripts, which have been reported to accompany aging (Meshorer and Soreq, 2002; Deschenes and Chabot, 2017; Bhadra et al., 2020) did not reveal any differences until 20 months of age at which time liver transcripts from *Myc*KO mice contained ∼3-fold more non-canonically spliced transcripts than those of wild-type livers. It was speculated that, like the above-described heterogeneous causes of DNA damage, splicing defects would not only be another sign of premature aging but might also contribute to the highly mutagenic environment of the *Myc*KO background that could be a major contributor to the aging and pro-senescence phenotypes the cells from these animals (Koh et al., 2015; Deschenes and Chabot, 2017; Bhadra et al., 2020; Wang et al., 2022b; Wang et al., 2023).

Several features of the gene expression differences between young and old wild-type and *Myc*KO mice lent further credibility to the notion that the premature aging of the latter actually represented an acceleration of otherwise normal processes. First, the gene set differences between the 2 groups were greater in the young mice than in older mice. This suggested that the same transcriptional changes were occurring between the 2 groups except that they accumulated faster in the *Myc*KO group. Second, the previously noted “universal” 79 member collection of age-related genes was less dysregulated between the 20 month old groups of mice than it was between the 5 month old groups. The changes in expression were somewhat different for this gene set than for the previously described transcript sets associated with Types 1 and 2 diabetes and cancer where the differences between the wild-type and *Myc*KO groups were detected in the youngest mice and the numbers and identities of the gene set transcript members changed somewhat between the younger and older cohorts.

The remarkable degree to which a broad range of age-related phenotypes and genes was altered in both *Myc* ± and *Myc*KO mice suggested that these models were more fully integrated into the physiologic networks that oversee normal aging than were the monogenic disorders associated with DNA damage recognition and repair that are commonly used as models of aging (Kalb et al., 2006; Brosh and Bohr, 2007; Opresko and Shay, 2017; Rizza et al., 2021). Thus, using data from their own *Myc*KO mice, the ENCODE and *Tabula Muris* Consortia and elsewhere, Wang et al. next focused on Myc and its direct target genes in an assortment of tissues from normal aging mice and humans (Encode Project Consortium, 2012; Diehl and Boyle, 2016; Tabula Muris Consortium, 2020; Wang et al., 2023). Initially using RNAseq results from young and old normal mice, they first identified highly significant age-related declines in Myc expression in 12 of 90 single-cell populations isolated from 23 different tissues. Even more impressive declines in more than 60% of direct Myc target gene sets were seen in one or more of the single cell populations from most of the above tissues. In those cases where the directionality of gene expression could be ascertained, it was highly correlated with age-related reductions in Myc. These studies thus documented age-related loss of Myc expression in normal aging mouse tissues and even more extensive effects on direct Myc target genes.

Myc levels in *in vitro* propagated primary human fibroblasts decline with time, and the inevitable onset of senescence and growth inhibition can be delayed or reversed by sustaining Myc expression (Dean et al., 1986; Benanti et al., 2007; Wang et al., 2022b). In addition, a previous study with cultured primary fibroblast samples from >650 young and old humans had shown that replicative senescence occurred more rapidly in the latter group (Trumpp et al., 2001; Smith et al., 2002; Wang et al., 2008; Wang et al., 2022b). Based on the above findings, Wang et al. therefore examined RNAseq data from a large number of normal human tissues from the Broad institute’s GTEx data base and divided these into young and old cohorts (∼20–40 years of age vs.∼60–80 years of age). Although variable, Myc levels were on average lower in several aged tissues, most notably sigmoid colon, adipose tissue and peripheral leucocytes. As was true for murine tissues, a collection of direct Myc target gene sets from the MSigDB data base showed that the expression of positive Myc targets declined in older tissues and negative Myc targets increased.

**FIGURE 2:**
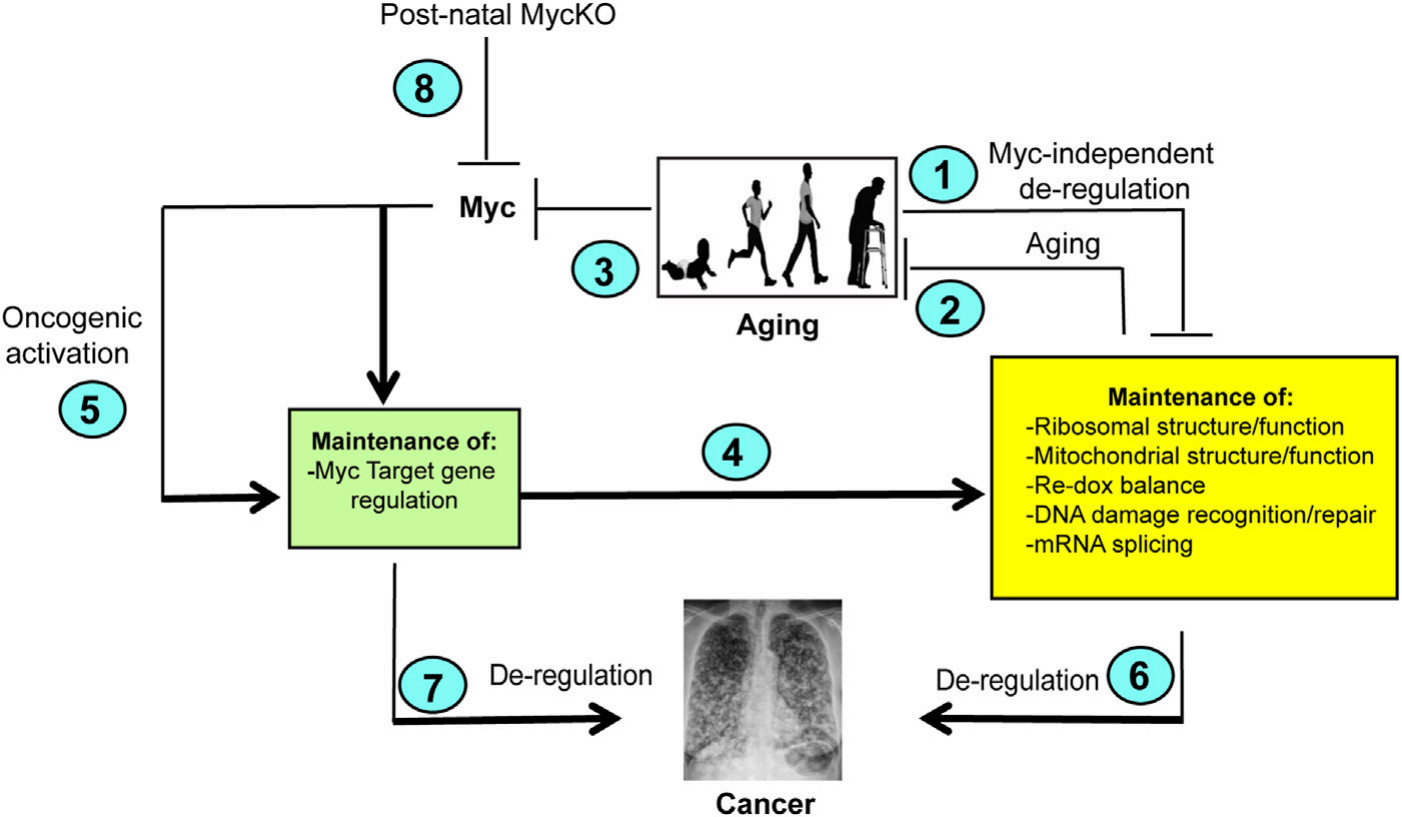
Model depicting the cooperation between normal aging and Myc. (1). Normal aging is associated with the accumulation of Myc-independent defects in a number of important cellular functions and/or an inability to maintain the regulation associated with youth. Examples include losses in translational efficiency, impaired mitochondrial function, increased ROS production, the accumulation of DNA damage and splicing (Meshorer and Soreq, 2002; Balaban et al., 2005; Park and Gerson, 2005; Short et al., 2005; Gonskikh and Polacek, 2017; Opresko and Shay, 2017). In turn, these functions may impact one another. For example, the high levels of ROS generated as a result of mitochondrial dysfunction can inhibit translation and induce oxidative DNA damage (Kirkinezos and Moraes, 2001; Vafa et al., 2002; Prochownik and Li, 2007; Rosca et al., 2012; Ghosh and Shcherbik, 2020; Molenaars et al., 2020). (2). The above-mentioned functions are also needed to maintain normal rates of aging. For example, defined defects in mitochondrial function and DNA damage recognition/repair pathways can accelerate aging (Opresko and Shay, 2017; Hahn and Zuryn, 2019; Rizza et al., 2021; Miwa et al., 2022; Shcherbakov et al., 2022). (3). Normal aging is associated with gradual declines in Myc and the ensuing dysregulation of Myc target gene expression (green box) (Wang et al., 2023). (4). As a result of declining Myc levels (3), normal aging leads to gradual declines in the expression of positively-regulated Myc target genes and increases in the expression of negatively-regulated Myc target gene (green box) (Wang et al., 2023). (5). Oncogenic activation of Myc deregulates its target genes leading to the constitutive up- or downregulation of its target genes (Figure 1), thereby driving increases in ribosome content, translation, mitochondrial mass and function, ROS production and DNA damage (Coller et al., 2000; Vafa et al., 2002; Felton-Edkins et al., 2003; Dang et al., 2006; Prochownik and Li, 2007; Prochownik, 2022). (6) The dysregulation of Myc target genes shown in (5) stabilizes or reverses the normal age-related changes in their expression and instead can drive and/or support the cellular processes necessary to maintain high levels of cancer-associated gene expression. (Shachaf et al., 2004; Dolezal et al., 2017; Prochownik, 2022). (7). Myc target genes not necessarily included in the yellow box, such as those which maintain cell cycle and impair apoptosis and senescence may independently contribute to tumor evolution when they are dysregulated as a result of Myc over-expression (Dang, 2011, 2012; Gabay et al., 2014; Prochownik, 2022). Some of these are likely to be “pathological targets” with low-affinity Myc binding sites and are activated only Myc levels exceed a certain threshold (Prochownik, 2022; Prochownik and Wang, 2022). (8). *Myc*KO mice fail to properly regulate their target genes. They therefore lose the ability to maintain the functions depicted in the yellow box. This lead to an accelerated aging phenotype, particularly in collaboration with the normal age-related declines in Myc-independent function (2). At the same time, the los of Myc eliminates major oncogenic pathways (4,5, and 7) thereby leading to an overall lifetime reduction in cancer susceptibility that contributes to longer survival even in the face of co-morbidities that are normally associated with shorter lifespans such as lipid accumulation and defective DNA damage recognition and repair. Created with BioRender.com.

Taken together, the studies of Wang et al. demonstrate that the abnormal findings associated with body-wide *Myc* knockout initiated shortly after birth are the consequence of a combination of the loss of this gene, dysregulation of its direct targets and the normal aging process (Wang et al., 2023). As such, these results and the previous ones obtained from *Myc* ± mice allow for the conclusion that Myc and its downstream target genes oversee the timing of many if not all of the most important aspects of normal aging (Figure 2) (Hofmann et al., 2015). It also showed that many of the findings previously associated with tissue-specific *Myc* inactivation could be recapitulated with body-wide knockout that included multiple tissue components rather than just a single one (Pshenichnaya et al., 2012; Edmunds et al., 2016; Wang et al., 2018). On the other hand, some of the most pronounced findings associated with *Myc* ± *and Myc−/−* mice such as growth retardation and smaller body size were not seen in *Myc*KO mice indicating that these phenotypes are determined prior to birth and that the substantial growth that occur post-natally is much less impacted by *Myc* loss (Davis et al., 1993; Trumpp et al., 2001; Dubois et al., 2008). On the other hand, even the low levels of Myc expression in some tissues of *Myc*KO mice may have been sufficient to rescue some of the more severe consequences that have been attributed to the total *Myc*KO and that is achievable only with embryonal targeting.

Aging and cancer are intimately linked, with advanced age being among the strongest predictors of cancer development (Pettan-Brewer and Treuting, 2011; White et al., 2014; Snyder et al., 2016). This relationship is particularly notable among individuals with monogenic disorders of DNA damage recognition and repair, known as progeroid syndromes, who despite their young chronological age, show signs of pronounced premature aging that can be reproduced in animal models (Blasco, 2005; Park and Gerson, 2005; Brosh and Bohr, 2007; Knoch et al., 2012; Opresko and Shay, 2017; Folgueras et al., 2018; Rizza et al., 2021; Rossiello et al., 2022). These disorders resemble normal aging in the sense that “aging” and the predisposition to cancer remain connected phenotypically if not chronologically. In contrast, a possible lower incidence of cancer in *Myc* ± mice described by Hofmann et al. (2015), might have been due to their overall healthier life span, such that this aspect of aging and cancer remained phenotypically linked as well. *Myc*KO mice, with a more than 3-fold lower cancer incidence despite their premature aging and increased lifespans, therefore represent a unique example in which chronological age and cancer incidence can be genetically separated and attributed to a single gene, namely *Myc.* The inextricable association between Myc and its role in driving and/or maintaining cancer, even when it is not needed to initiate tumors, likely explains the significantly lower cancer incidence of *Myc*KO mice (Karlsson et al., 2003; Wu et al., 2007; Meyer and Penn, 2008; Stine et al., 2015; Dolezal et al., 2017; Wang et al., 2023). This is underscored by the observation that the rare tumors that did arise in these animals tended to express Myc and contained at least a diploid or pseudo-diploid *Myc* DNA content, indicating that cells with incomplete *Myc* excision were selected for neoplastic transformation in aged individuals. Interestingly, an example of a human progeria syndrome that is not associated with a high incidence of cancer early in chronological life is Hutchinson-Guilford progeria (HGP), which is caused by mutations in the laminin A (*LMNA*) gene (Sarkar and Shinton, 2001; Sinha et al., 2014). While these individuals do show evidence of genomic instability and defective DNA repair, the primary *LMNA* mutations in HGP cause an abnormal nuclear architecture and loss of heterochromatin organization and its contact with the nuclear envelope (Arancio et al., 2014). We examined the catalogued RNAseq data from 2 studies that analyzed the differences between HGP and normal human fibroblast transcriptomes and found no evidence for the dysregulation of Myc or its target genes (Kohler et al., 2020; San Martin et al., 2022). Nor did the authors of these reports identify irregularities in the expression of any of the major Myc target gene categories. Thus, although aging and cancer can be dissociated in HGP as it can in *Myc*KO mice, it appears unrelated to any changes in the expression of *Myc* or its target genes.

## 5 The *Myc*KO mouse as a new (and improved?) model for premature aging?

**FIGURE 3:**
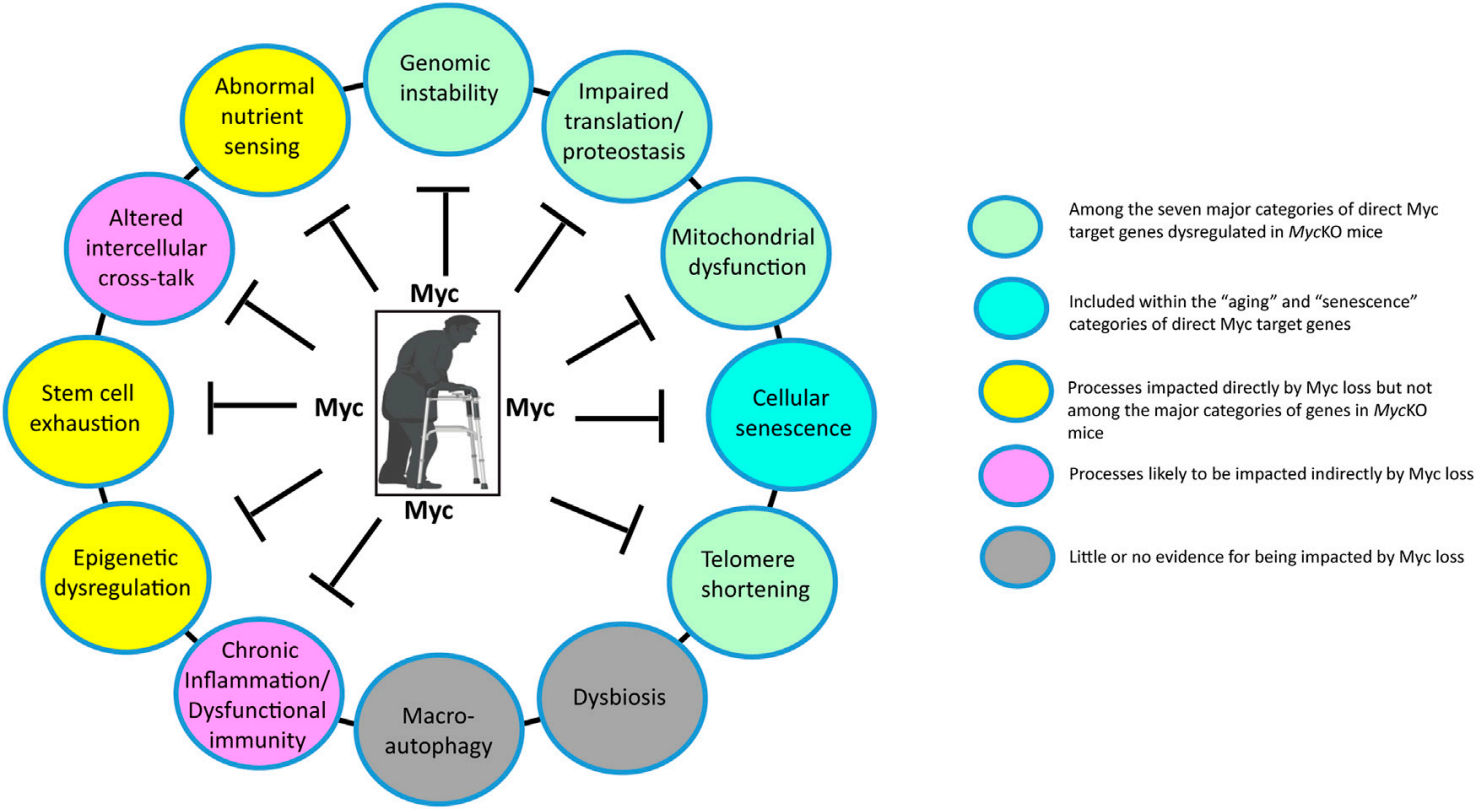
The impact of Myc on the Hallmarks of Aging. Depicted here are the major molecular, cellular and whole body changes that represent the common denominators of aging and how they are impacted by Myc (Lopez-Otin et al., 2023). Many previously described direct Myc target genes are involved in maintaining the structure and function of ribosomes, translation factors and mitochondria (Li et al., 2005; Gomez-Roman et al., 2006; Ruggero, 2009; Morrish et al., 2010; van Riggelen et al., 2010; Morrish and Hockenbery, 2014; Dolezal et al., 2017; Singh et al., 2021; Prochownik, 2022). Myc also regulates glutamine metabolism and its anaplerotic entry into the TCA cycle, while also promoting glycolysis by directly up-regulating the genes encoding most enzymes in the glycolytic pathway, particularly the rate-limiting ones (Dang, 2011; Stine et al., 2015; Prochownik, 2022; Prochownik and Wang, 2022). Both the over- and under-expression of Myc can promote genomic instability via the regulation of genes involved in DNA damage recognition and repair, telomere maintenance, the generation of genotoxic ROS and the promotion of tetraploidy (Yin et al., 1999; Vafa et al., 2002; Prochownik and Li, 2007; Wang et al., 2022b; Solvie et al., 2022; Wang et al., 2023). Myc’s transcriptional control over genes involved in mRNA splicing, together with Myc-induced genomic instability, can contribute to a higher background of neo-antigen production and inflammation while altering rates of aerobic and anaerobic respiration by, for example, altering splicing choices for genes such as that encoding pyruvate kinase (Meshorer and Soreq, 2002; David et al., 2010; Koh et al., 2015; Wang et al., 2022b; Prochownik, 2022; Wang et al., 2023). Myc can also suppress senescence and maintain the stem cell niche (Dean et al., 1986; Wu et al., 2007; Dubois et al., 2008; Zhuang et al., 2008; Vilas et al., 2018) and can also impact intercellular communication by regulating the expression of cytokines, chemokines and immune checkpoints (Hayashi et al., 1998; Yi et al., 2003; Singh et al., 2006; Piddock et al., 2018). Myc’s control over its target genes largely involves epigenetic re-programming, primarily at the level of histone H3/H4 acetylation and/or methylation (Knoepfler et al., 2006; Kalkat et al., 2018; Tu et al., 2018). Finally, Myc may be involved indirectly in the regulation of macroautophagy given that Miz1 appears to be involved in this process (Wolf et al., 2013). Created with BioRender.com.

Mouse models of the above-discussed progeroid syndromes have long been used as surrogates for normal human aging (Harkema et al., 2016; Koks et al., 2016; Folgueras et al., 2018; Rizza et al., 2021). However, these models are based on exceedingly rare monogenic disorders that are of questionable relevance to normal aging aside from recapitulating some of its associated phenotypes. This is because they directly impact only one or 2 of aging’s so-called “Hallmarks”, namely those pertaining to genomic stability and telomere maintenance (Figure 3) (Lopez-Otin et al., 2023). Thus they likely over-emphasize the roles of these 2 hallmarks while discounting the roles of others. While the *Myc*KO model is also monogenic, it differs importantly from progeroid syndrome models primarily because the loss of Myc, which is a transcription factor, is more consequential by virtue of directly impacting the vast majority of aging’s hallmarks (Figure 2). As discussed above, for example, 4 of the 7 major categories of gene sets that are impacted in *Myc*KO mice and MEFs (i.e. ribosomal/mitochondrial structure and function, DNA damage response/repair and splicing directly impact 4 of the Aging Hallmarks (Figure 3) (Meshorer and Soreq, 2002; Short et al., 2005; Tchkonia et al., 2010; D'Aquila et al., 2017; Deschenes and Chabot, 2017; Gonskikh and Polacek, 2017; Bhadra et al., 2020). The categories pertaining to senescence and aging are directly related to 2 additional Aging Hallmarks and the dysregulation of the transcripts within these categories reflects the declines of Myc and its direct target genes that accompany normal aging in both mice and humans (Dean et al., 1986; Smith et al., 2002; Benanti et al., 2007; Wang et al., 2023). In addition, the category pertaining to oxidative stress, which is associated with high levels of ROS and ROS-mediated damage in *Myc*KO cells and tissues (Edmunds et al., 2016; Wang et al., 2022b) can accelerate the onset of other Aging Hallmarks, including genomic and mitochondrial DNA instability and impaired translation (Gonskikh and Polacek, 2017; Hahn and Zuryn, 2019; Payea et al., 2021; Renaudin, 2021; Woodward and Shirokikh, 2021; Wang et al., 2022b). While gene categories involved in nutrient sensing, stem cell maintenance and epigenetic regulation were not among the top ones identified in the tissues of *Myc*KO of mice (Wang et al., 2022b; Wang et al., 2023), Myc has long been known to play important roles in these processes; indeed Myc positively regulates transcription via its ability to recruit epigenetic modifiers to its bound sites in chromatin (Amati et al., 2001; Ba et al., 2018; Kalkat et al., 2018). Moreover, its age-related decline is likely to play some role in determining the pace at which these functions deteriorate (Figure 3) (Chappell and Dalton, 2013; Kalkat et al., 2018; Prochownik and Wang, 2022). Finally, 2 of the Aging Hallmarks pertaining to intercellular communication and chronic inflammation are likely to be indirectly regulated by Myc, which controls the expression of a number of cytokines, chemokines and immune checkpoints that contribute to these processes (Hayashi et al., 1998; Yi et al., 2003; Singh et al., 2006; Casey et al., 2018; Piddock et al., 2018). The Myc-dependent re-organization of glucose and glutamine metabolism has been shown to be essential for the expansion of activated T cells (Dang, 2010; Wang et al., 2011).

The wide-ranging consequences of *Myc* inactivation make it highly unlikely that, as is true for normal aging, its impact on any single Aging Hallmark can fully explain the premature aging profile of *Myc*KO mice (Figure 3). Just as genotoxic damage and telomere attrition drive some aspects of premature aging in progeria syndromes, so too can the interference with protein synthesis, mitochondrial DNA integrity and stem cell regulation, all of which are Myc-dependent to varying degrees (Hiona and Leeuwenburgh, 2008; Vilas et al., 2018; Shcherbakov et al., 2022). While the general categories of gene sets that are dysregulated in *Myc*KO mice are similar, the degree to which their component transcripts are enriched as well as their individual identities differ among the limited number of tissues and cell types that have been thus far surveyed, namely MEFs, liver, skeletal muscle and adipose tissues (Wang et al., 2022b; Wang et al., 2023). These seemingly subtle distinctions may well exert significant influence over which tissues display signs of premature aging, what these signs are, when they first appear and their severity. Finally, it should be kept in mind that, unlike the generation of *Myc−/−* and *Myc* ± mice, which provide consistent levels of *Myc* knockout in each tissue (Davis et al., 1993; Trumpp et al., 2001; Hofmann et al., 2015), the degree of Myc loss in *Myc*KO mice varies both among and within tissues and individual mice, while also showing some age-related recovery (Wang et al., 2023). Indeed, even the 80%–90% levels of knockout routinely achieved may still be sufficient to allow normal or near-normal regulation of certain direct target genes with the highest affinity Myc binding sites. Considerable growth and development normally continue beyond the time of weaning that marks the point in time of *Myc* gene deletion (Wang et al., 2023). It thus remains to be determined whether even higher gene knockout efficiencies would have remained compatible with the prolonged survival noted by Wang et al. and whether other phenotypes might have emerged.

Yet to be fully explained is why mice with a 50% normal level of Myc and those with ∼80->90% knockout have such different aging phenotypes (Hofmann et al., 2015; Wang et al., 2023). Here too there may be no single answer but at least 2 major and non-mutually exclusive reasons may pertain to the timing of *Myc* gene knockout and dose-related effects. The first may be related to the fact that, when *Myc* inactivation is initiated in the embryo, gene dose determines the resultant body size whereas when Myc is inactivated post-natally, no effect on overall body size is observed (Trumpp et al., 2001; Hofmann et al., 2015; Wang et al., 2023). The relationship between body size and longevity is well-known, with smaller members of the same species ranging from flies to humans tending to have longer average lifespans (Samaras et al., 2003; Khazaeli et al., 2005; Blagosklonny, 2013). Whether the increased longevity of *Myc* ± mice arises simply as a consequence of their smaller body size and is thus not a direct *Myc* gene dosage effect could be determined by engineering a precise 50% knockout of *Myc* post-natally so as to genotypically mimic *Myc* ± mice while avoiding the size disparities previously noted when haplo-insufficiency is generated in the embryo (Hofmann et al., 2015). The second reason pertaining to gene dosage involves the phenomenon of “heterozygous advantage” whereby possessing a single mutant or inactive allele can confer a selective survival advantage whereas mutational homozygosity can be deleterious or even lethal (Hedrick, 2012). Examples of such genes include those encoding the cystic fibrosis transmembrane conductance regulator, the α- and β-globins and triose phosphate isomerase (Cuthbert et al., 1995; Jones, 1997; Destro-Bisol et al., 1999; Ralser et al., 2006).

## 6 Questions for the future

The studies reviewed, compared and summarized here have clearly indicated that Myc plays a significant role in balancing the overall health and wellness of mice and probably of humans as well. Myc is also involved in the development and timing of tumors that appear to impact their natural life spans. However, a number of questions remain unanswered. For example, the high mortality rate associated with inactivating the *Myc* gene prior to about 1 month of age leaves open the question of its role in the considerable amount of growth and development that occurs prior to this time. The work of Wang et al. and the earlier work of Soucek et al. showed that the initial inhibition of *Myc*, even in adult mice, is accompanied by significant changes in the gastrointestinal and hematopoietic compartments (Soucek et al., 2008; Wang et al., 2023). What limits the severity of these changes and why is the latter so much more severe prior to weaning and particularly so in the embryo (Davis et al., 1993; Trumpp et al., 2001; Dubois et al., 2008)? Perhaps an even more fascinating question is what factor(s) contribute to the reversal of these initial changes and allow these highly proliferative tissues to remain so over the course of a lifetime in the face of little to no expression of Myc?

This last question remains particularly germane when considering the nearly universal requirement for Myc in maintaining the proliferation of non-transformed cells *in vitro* (Mateyak et al., 1997; Wang et al., 2022b). The critical contribution of Myc to maintaining rapid tumor cell growth and/or viability both *in vitro* and *in vivo* has also been demonstrated in many transformed cell types and a variety of neoplasms, including those arising in bone, the lymphatic system and the liver (Shachaf et al., 2004; Wu et al., 2007; Wang et al., 2008; Gabay et al., 2014; Dolezal et al., 2017). Yet, there are clear exceptions to this rule, most notably in the case of the liver where the short-term regeneration of the organ following partial hepatectomy and its longer-term repopulation by transplanted hepatocytes are entirely Myc-independent (Baena et al., 2005; Khazaeli et al., 2005; Sanders et al., 2012; Edmunds et al., 2016). In the hepatoblastoma model, where Myc is not one of the driver oncogenes, but is expressed at a high level, tumor initiation remains highly effficient in Myc’s absence although survival is markedly prolonged due to slower tumor growth (Wang et al., 2016). Although no comparative studies have been done, it would appear that, both *in vitro* and *in vivo*, Myc in many cases is required to maintain cellular growth at maximum rates, particularly for tumors and even more so for those tumors in which Myc is the actual driver oncogene. Cells that proliferate relatively slowly and are not transformed may therefore be less reliant on Myc to maintain this state.

The extent to which different functions that are impacted by *Myc*’s loss contribute to premature aging (Wang et al., 2023) also remains a major question. Impaired mitochondrial function, metabolism and translation have all been described in association with aging and all of these are dependent upon Myc to balance and maintain their normal function (Li et al., 2005; van Riggelen et al., 2010; Barrientos, 2012; Graves et al., 2012; Edmunds et al., 2016; D'Aquila et al., 2017; Gonskikh and Polacek, 2017; Miwa et al., 2022; Prochownik and Wang, 2022). The degree to which these drive the premature aging phenotypes of *Myc*KO mice may well be impacted by and synergize with one another. An example of this is the relationship between what are arguably the 2 major drivers of aging, namely ROS and DNA damage, which are inextricably linked by virtue of the fact that both Myc over- and under-expression can drive ROS production which in turn can cause oxidative DNA damage (Vafa et al., 2002; Balaban et al., 2005; Brosh and Bohr, 2007; van Riggelen et al., 2010). Virtually all the major pathways that are under Myc’s control are known to be associated with or to drive aging and senescence when they are deregulated (Figures 2, 3) (van Riggelen et al., 2010; Popay et al., 2021; Prochownik, 2022; Prochownik and Wang, 2022).

Two additional and related issues worth examining in future studies are whether the inactivation of Myc later in life also accelerates aging and whether normalizing Myc expression can reverse this process or at least “reset” the aging clock. These are important practical questions given the long-standing interest in inhibiting Myc as a general chemotherapeutic strategy for cancer (Llombart and Mansour, 2022). The finding that *Myc* ± mice showed both increased longevity and improved overall health initially suggested that the use of Myc inhibitors to treat various cancers might actually have additional secondary benefits and even hold promise as anti-aging therapies akin to those provided by caloric restriction or metformin (Kebbe et al., 2021). However, the more recent work of Wang et al. (2023) suggests that this might not be the case (at least in cancer) given that total Myc inhibition would be the desired therapeutic goal in treating cancer and would more likely than not accelerate rather than slow aging as one of its potential side effects. This consideration might have particular relevance for older adults who in many cases are already frail at the time they begin chemotherapy and who can ill afford to age any more rapidly (Ness and Wogksch, 2020; Shafqat et al., 2022). Perhaps even more deserving of consideration would be whether Myc inhibitors should be used in children in whom even relatively short courses of standard cancer chemotherapy can elicit features of premature aging and might collaborate with agents that deliberately lowered Myc levels (Smitherman et al., 2020; Kruseova et al., 2023). Similar concerns may be warranted over the use of such agents solely as potential lifespan and/or healthspan extenders or in the long-term treatment of non-malignant conditions associated with other Myc-dependent hyperproliferative states (Prochownik and Vogt, 2010).
